# Microglial lactate metabolism as a potential therapeutic target for Alzheimer’s disease

**DOI:** 10.1186/s13024-022-00541-z

**Published:** 2022-05-12

**Authors:** Yingjun Zhao, Huaxi Xu

**Affiliations:** 1 Center for Brain Sciences, the First Affiliated Hospital of Xiamen University, Institute of Neuroscience, Fujian Provincial Key Laboratory of Neurodegenerative Disease and Aging Research, Xiamen University, Xiamen, China; 2 Institute for Brain Science and Disease, Chongqing Medical University, Chongqing, China

Microglia are the major type of resident immune cells within the brain that exert immune protection under pathophysiological conditions by clearing away pathogens, cellular debris and misfolded proteins, thereby maintaining a clean and healthy microenvironment for the brain [1]. Accumulating evidence has shown that microglia are involved in the pathogenesis of Alzheimer’s disease (AD), but their exact role is still ambiguous [2]. Particularly, it has been shown that microglial metabolism and cellular functions are tightly linked [3]. However, how metabolic control of microglial functions affects the development and progression of AD remains unknown.

In a recent publication in *Cell Metabolism*, Pan et al. reported a glycolysis/H4K12 lactylation/PKM2 positive feedback loop in microglia that drives the pathogenesis of AD [4]. They showed that this vicious loop exacerbated glucose metabolism disorder and pro-inflammatory activation of microglia in AD, while breakdown of this loop could curb the development of AD pathology and cognitive decline, suggesting that inhibiting glycolysis/H4K12 lactylation/PKM2 loop in microglia is a potential therapeutic strategy for the treatment of AD.

Microglia utilize both glycolysis and oxidative phosphorylation (OXPHOS) for energy metabolism. Quiescent microglia are thought to primarily rely on OXPHOS for ATP production, whereas activated microglia display a metabolic switch phenotype from OXPHOS to glycolysis. This metabolic switch has been observed in several neurodegenerative diseases including Parkinson’s disease (PD) and AD. Increased glucose uptake in microglia was recently identified in AD mice and patients, and this metabolic state positively associated with the extent of neuroinflammation [5]. Consistently, Pan and colleagues found that the lactate levels dramatically increased in the microglia from a transgenic AD mouse model (5XFAD), suggesting that the increased microglial glycolysis contributes to the lactate metabolism disorder in the context of AD.

Lactate is not only a product of glycolysis, but also a substrate for histone lactylation that has been established recently [6]. This novel epigenetic modification was shown to directly regulate gene transcription and was associated with a serial of biological processes such as macrophage polarization, somatic cell reprogramming, and tumorigenesis [6–8]. Pan et al. showed that histone lactylation was markedly increased in the brain tissues from AD mice and patients, and H4K12la (Histone lactylation at H4 Lysine 14) was the most prevalent differentially altered epigenetic mark. They further revealed that H4K12la was specifically up-regulated in the amyloid-beta (Aβ)-associated microglia. Using the CUT&Tag technique, they demonstrated that H4K12la was enriched at the promoters of glycolytic genes (*i.g.*, *Pkm* and *Ldha*) and activated transcription of these genes, thus forming a “glycolysis-lactate-histone lactylation-glycolysis” positive feedback loop and increased the glycolytic activity.

Sustained activation of glycolytic metabolism would lead to low efficiency of ATP production and compromise of microglial immune functions [9]. Since the energy metabolism is required for Aβ phagocytosis and clearance, the low efficiency of ATP production due to glycolytic metabolism would impair the phagocytic function of microglia and result in Aβ accumulation. In addition, glycolytic phenotype and lactate accumulation in microglia could promote the production and release of pro-inflammatory cytokines, resulting in chronic neuroinflammation, neuronal damage, and thus the development and progression of AD. Strikingly, Pan et al. showed that interruption of the glycolysis/H4K12 lactylation/PKM2 loop by pharmacological or genetic approaches inhibited microglial activation, reduced Aβ pathology, and improved cognitive function of AD mice.

In summary, this study highlights a crosstalk between lactate metabolism and histone lactylation in microglia, and reveals how this lactate-derived epigenetic modification exacerbates microglial dysfunction and neuroinflammation in the development and progression of AD. Therefore, targeting lactate metabolism disorder may represent a novel strategy for AD intervention (Fig. 1). It will be also interesting to investigate whether and how other signaling pathways mediating microglial metabolism such as TREM2 pathway are involved in the crosstalk between lactate metabolism and histone lactylation in the context of AD [10].

**Fig. 1:**
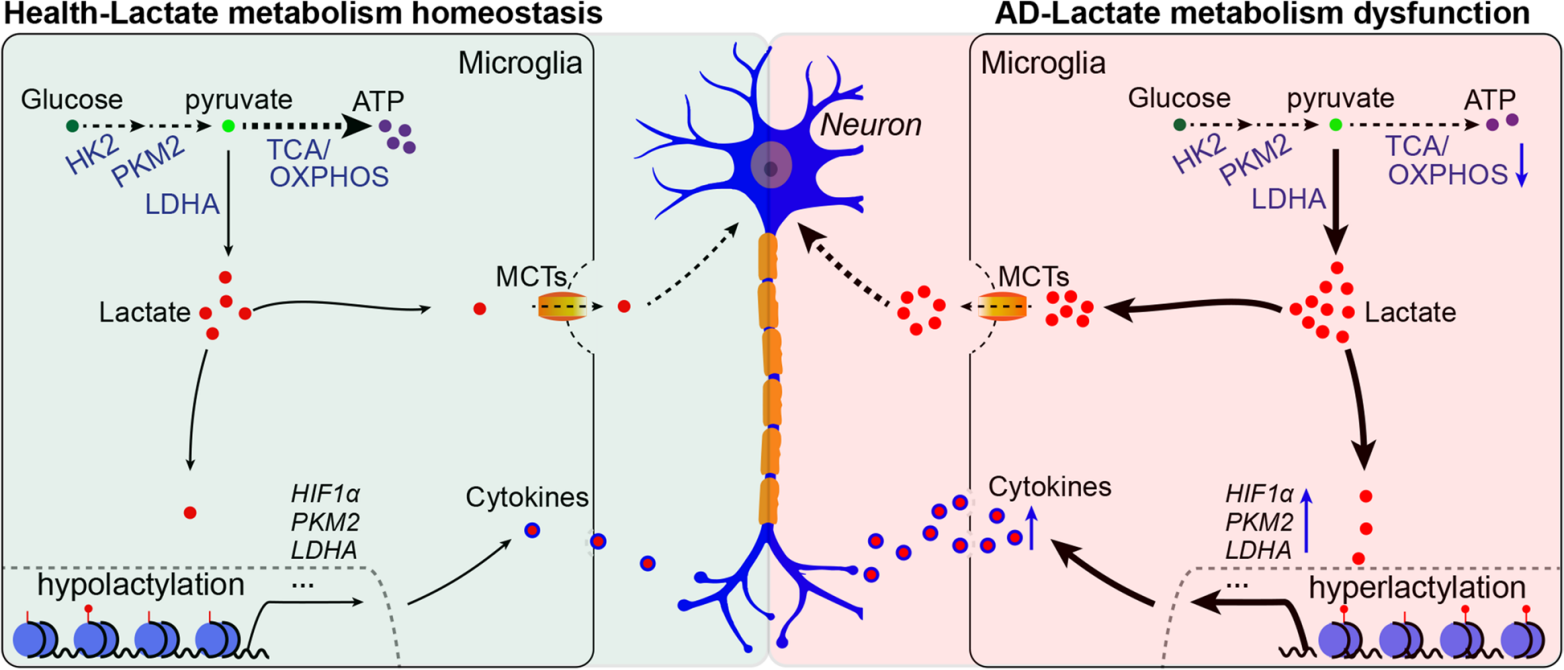
Lactate metabolism disorder in microglia of Alzheimer’s disease. Under the healthy condition, microglia maintain the homeostasis of lactate metabolism. Along with aging or AD development, microglia up-regulate glycolytic machinery and switch metabolism from OXPHOS to aerobic glycolysis, resulting in lactate accumulation and histone hyperlactylation. On the one hand, lactate is released to the extracellular through monocarboxylate transporters (MCTs) and affects the acidity of microenvironment, leading to neuronal damage; on the other hand, lactate is transported to the nuclei and leads to histone lactylation in turn promotes glycolytic activity through transcriptional activation of glycolytic genes, thus exacerbating lactate metabolism disorder and neuroinflammation during AD pathogenesis

## Data Availability

Not applicable.

## Abbreviations

AD: Alzheimer’s disease
Aβ: Amyloid-beta
OXPHOS: Oxidative phosphorylation
PD: Parkinson’s disease
